# Enhanced anti-cancer activity of andrographis with oligomeric proanthocyanidins through activation of metabolic and ferroptosis pathways in colorectal cancer

**DOI:** 10.1038/s41598-021-87283-y

**Published:** 2021-04-06

**Authors:** Tadanobu Shimura, Priyanka Sharma, Geeta G. Sharma, Jasjit K. Banwait, Ajay Goel

**Affiliations:** 1 Center for Gastrointestinal Research, Baylor Scott & White Research Institute and Charles A. Sammons Cancer Center, Baylor University Medical Center, Dallas, TX USA; 2 Department of Molecular Diagnostics and Experimental Therapeutics, Beckman Research Institute, City of Hope Comprehensive Cancer Center, Duarte, CA USA; 3 Department of Molecular Diagnostics and Experimental Therapeutics, Biotech Innovations, Beckman Research Institute, City of Hope Comprehensive Cancer Center, 1218 S. Fifth Avenue, Suite 2226, Monrovia, CA 91016 USA

**Keywords:** Cancer, Oncology

## Abstract

The high degree of morbidity and mortality in colorectal cancer (CRC) patients is largely due to the development of chemoresistance against conventional chemotherapeutic drugs. In view of the accumulating evidence that various dietary botanicals offer a safe, inexpensive and multi-targeted treatment option, herein, we hypothesized that a combination of *Andrographis paniculata* and Oligomeric Proanthocyanidins (OPCs) might interact together with regard to anti-tumorigenic activity in CRC. As a result, we demonstrated the enhanced anti-cancer activity between these two botanical extracts in terms of their ability to inhibit cancer cell growth, suppress colony formation and induce apoptosis. Furthermore, we validated these findings in subcutaneous xenograft model and in patient derived primary epithelial 3D organoids. Transcriptomic profiling identified involvement of metabolic pathways and ferroptosis-associated genes, including HMOX1, GCLC and GCLM, that may be responsible for the increased anti-tumorigenic activity by the two compounds. Collectively, our study provides novel evidence in support of the combinatorial use of andrographis and OPCs as a potential therapeutic option, perhaps as an adjunctive treatment to classical drugs, in patients with colorectal cancer.

## Introduction

Colorectal cancer (CRC) is the third most common cancer worldwide^1^. Systemic chemotherapy remains one of the hallmark treatments for metastatic CRC (mCRC) patients; even though a significant majority of these patients tend to develop resistance to such treatment due to inherent or acquired development of chemo-resistance. Although 5-fluorouracil (5-FU)-based chemotherapies still remain the key-treatment in mCRC patients^2^, the effectiveness of these treatments often reduce mainly coursed by chemo-resistance, which is a complex multistep process that manifests because of dysregulated expression of key growth regulatory genes and altered cellular signaling in associated pathways^3^. To muddy the things further, acquired resistance to first-line treatment often confers resistance to subsequent lines of therapies, leading to the concept of multidrug resistance (MDR)—often the Achilles heel of cancer therapy in various human malignancies^4^. While there are no effective answers to overcome MDR, data in the recent years have provided an unequivocal evidence that use of a single-agent cancer therapy, especially in advanced cancers, is very unlikely to be effective in eradicating a tumor. Hence, a multimodality, combination treatment that encompasses the use of multiple drugs which influence multiple growth signaling pathways might offer a more attractive and effective therapeutic option in cancer patients^5^. In this context, a recent study emphasized the need for developing more informed anti-cancer therapeutic regimens that target distinct cancer pathways simultaneously, instead of their sequential administration, which is often inadequate but still exposes the patient to toxicity and expense of these drugs^6^. Furthermore, evidence suggests that the use of multi-targeted therapies frequently leads to reduced drug dosages when used in combination vs. their individual concentrations—in most instances with fewer deleterious side-effects and dose-limiting toxicities^7^.

Accumulating evidence indicates that certain dietary agents such as curcumin, green tea, boswellia, grape seed extract, and andrographis possess anti-tumorigenic properties by targeting multiple oncogenic signaling pathways^8–17^. Andrographolide is one of the principle diterpenoid lactones isolated from the traditional herb *Andrographis paniculata*, which possesses various biological activities, including anti-inflammatory, immunomodulatory, antiviral, and antitumoral effects, with a wide-ranging use for the treatment of sore throat, fever, diarrhea, and inflammatory disorders including colitis^18^. In recent years, increased number of studies have revealed that andrographis treatment leads to inhibition in cell proliferation and high rates of apoptosis in a variety of human cancer cells. Such observations were further supported by its ability to also regulate oxidative stress, cell cycle arrest, necrosis, autophagy, inhibition of cell adhesion, migration, invasion and anti-angiogenic activity^19^. In a similar context, research from our group and others have demonstrated the anti-tumorigenic properties of oligomeric proanthocyanidins (OPCs), a group of flavonoids present in the grape seed extract, to suppress CRC cell growth through other unique cell signaling pathways including self-renewal, drug-transporter system and metabolic pathways including protein export, glutathione metabolism and porphyrin metabolism^10, 17, 20^. Given the fact that a most effective anti-cancer treatment approach must be multi-targeted in nature, and because both andrographis and OPCs tend to exhibit their cancer inhibitory activity through distinct pathways, we hypothesized that a combination treatment with these botanical extracts might offer an enhanced anti-tumorigenic activity in CRC. This hypothesis was further supported by the school of thought that such dietary compounds are generally quite safe and inexpensive—providing a rationale for evaluating their combinatorial efficacy, vis-à-vis their anti-cancer activity individually.

While previous studies have interrogated the efficacy of andrographis on specific pathways in cancer cells^21, 22^, we for the first time performed a series of systematic cell culture, xenograft model, and patient-derived tumor organoid experiments to evaluate the combined anti-tumorigenic effect of andrographis and OPCs, and performed a whole genome transcriptomic profiling to find out key pathways involved in the combined treatment of andrographis and OPCs in colorectal cancer.

## Materials and methods

### Cell culture and materials

Colorectal cancer cell lines, HCT116 (MSI, KRAS-G13D mutant, BRAF wild type), HT29 (MSS, KRAS wild type, BRAF V600E mutant), and normal colonic epithelial cell; NCM460^23^ were purchased from the American Type Culture Collection (Manassas, VA). All cell lines were tested and authenticated using a panel of genetic and epigenetic markers and tested for mycoplasma on a regular basis. The cells were grown in Iscove’s Modified Dulbecco’s Media (IMDM; Gibco, Carlsbad, CA), supplemented with 10% fetal bovine serum, 1% penicillin and streptomycin and maintained at 37 °C in a humidified incubator at 5% CO2. Grape seed OPCs (VX1 extract; dissolved in phosphate buffered saline), and andrographis extract (EP80 andrographis extract standardized to 80% andrographolide content; dissolved in DMSO) were provided as a generous gift by EuroPharma-USA (Green Bay, WI, USA). Both compounds were diluted to appropriate experimental concentrations in culture medium.

### Cell viability, apoptosis and colony formation assay

Cells were plated in 96-well dishes at a density of 3000 cells/well in IMDM supplemented with 10% FBS and antibiotics and allowed to attach overnight. Cell proliferation was measured in cells treated with a combination of OPCs (30, 60, 90, 120, and 150 ug/ml) and andrographis (7.5, 15, 22.5, 30, and 37.5 ug/ml) for 72 h using WST-1 assay (Sigma-Aldrich, St. Louis, MO). We used uniform DMSO concentrations between each treatment group, including the untreated group. In order to assess the synergism between andrographis and OPCs in CRC cells, the combination index (CI) was calculated using the Chou–Talalay equation ^24^ at 50% inhibitory concentration using GraphPad Prism Ver.6.0 (GraphPad Software Inc., San Diego, CA). A CI index of less than 1 was considered to be a synergistic interaction. Each experiment was performed as three independent technical triplicates.

For the apoptosis assays, cells were plated in 6-well dishes, followed by treatment with OPCs, andrographis, and their combination, for 48 h. The apoptotic cell fraction was measured using a Muse Annexin V and Dead Cell Assay Kit (MCH100105, Millipore, Chicago, IL) on a Muse Cell Analyzer (Millipore) according to the manufacturer’s instructions.

For colony formation assay, 300 cells/well were used and the assays were performed as described previously^25^. All experiments were conducted as three independent technical triplicates.

### Genomewide transcriptomic profiling and pathway enrichment analysis

Total RNA was isolated from 2 × 10^6^ HCT116 and HT29 cells following treatment with each agent for 16 h. The RNA was isolated using MiRNeasy Mini kit (Qiagen, Hilden, Germany) according to manufacturer’s protocol. We extracted RNA from two independent wells in each treatment group from each cell-line. RNA extracts with RNA integrity number (RIN) values greater than 9.5 were used for gene expression profiling studies, using the ClariomS human gene expression arrays as described previously^26^. Probe set selection was performed using Detected Above Background (DABG) values of 0.05 or less, in more than 50% of samples. The raw expression data was preprocessed using Robust Multi-Array Average (RMA) method in Transcriptome Analysis Console (TAC 4.0.1). Differential expression analysis was performed to identify significantly (p ≤ 0.05) and differentially expressed genes (DEGs) using ebayes, using a threshold of p < 0.05 and |log FC| greater than 1. For the identified DEGs, we performed Gene Ontology (GO) and KEGG pathway enrichment analysis using the R package ‘cluster Profiler’, and gene sets with a p-value of less than 0.05 were considered significantly enriched. In order to construct gene expression profiling heatmaps, we used a R library Non-negative matrix factorization (NMF), which is an unsupervised learning algorithm that detects context dependent patterns of gene expression in complex biological systems^27, 28^.

### Quantitative mRNA expression analysis

RNA extraction from the cell lines, xenograft animal tumors, and CRC-derived organoids, treated with DMSO (vehicle), OPCs, andrographis and their combination, was performed using the MiRNeasy Mini Kit (Qiagen); followed by conversion to cDNA using the High Capacity cDNA Reverse Transcription Kit (ThermoFisher Scientific, Waltham, MA) as previously described^17^. cDNA derived from 5 ng of RNA was used and qRT-PCR was performed using SensiFAST SYBR mix (Bioline, London, UK) using the primer sequences listed in Supplementary Table S1. Briefly, cDNA samples were mixed with 0.5 µl of 10 µM each of forward and reverse primers specific for the target genes, 5 µl SYBR green master mix and volume was made up with nuclease-free water. The relative expression for target genes was calculated using 2^−ΔΔCT^ method normalized against the housekeeping β-actin gene.

### Western immunoblotting

For western immunoblotting experiments, tumor xenografts were lysed using the RIPA buffer supplemented with proteinase inhibitors (Bio-Rad Laboratories, Hercules, CA), which contained 5% 2-Mercaptoehanol (Sigma-Aldrich). The primary antibodies used were mouse monoclonal antibody γ-GCLM (sc-55586; Santa Cruz Biotechnology, Dallas, TX), rabbit polyclonal GCLC antibody (ab53179, Abcam, Cambridge, UK) and mouse monoclonal antibody HMOX1 (sc-136960, Santa Cruz Biotechnology). Anti-mouse IgG or anti-rabbit IgG secondary antibodies were purchased from Santa Cruz Biotechnology. A monoclonal mouse β-actin antibody (A5441, Sigma-Aldrich) was used as the loading control. Chemiluminescence images were obtained using ChemiDoc-MP Imaging system (ver 5.2.1, BioRad Laboratories Inc, Hercules, CA), and the band intensities were quantified using the Image J software ver. 1.52 (NIH, Bethesda, MD).

### Xenograft animal model

Seven-week-old male athymic nude mice (Envigo, Houston, TX) were housed under controlled light conditions and were provided with food and water ad libitum. Xenograft tumors were generated by subcutaneous injection of 1 × 10^6^ HCT116 cells. Tumor volume was calculated using the formula: (1/2) (length × width × height). Animals were randomly divided into four groups with 10 animals in each group: (1) untreated vehicle (PBS), (2) 50 mg OPC/kg body weight daily, (3) 125 mg andrographis/kg body weight every alternate day or (4) andrographis and/or OPCs together at the concentrations listed above. All treatments were injected intraperitoneally daily for 15 days, followed by euthanasia. Tumor samples were dissected, weighed and stored in RNAlater (Sigma-Aldrich) for further analysis. The animal protocol was approved by the Institutional Animal Care and Use Committee, Baylor Scott & White Research Institute, Dallas, Texas (Ethics code; A18-004, Approval date; 12/10/2018). All experiments were performed in accordance with relevant guidelines and regulations. The human equivalent dose was calculated using following formula as described previously^29^.

### Patient-derived tumor organoids

Fresh tumor tissues were obtained from CRC patients enrolled at the Baylor University Medical Center. The study was approved by the Institutional Review Board of Baylor Scott & White Research Institute, Dallas, TX. A written informed consent was obtained from all patients providing tissue specimens, and all experiments were performed in accordance with relevant guidelines and regulations proposed in the Declaration of Helsinki. CRC tumor organoids were cultured using a modified protocol described previously^17^. For treatments, appropriate concentration of OPCs and/or Andrographis were added to the culture medium and tumor organoids were grown for 1 week. The experiment was performed in technical replicates. The organoids were observed under a bright-field microscope. Organoids that were about 500 microns in diameter and in a certain plane of field were counted, while leaving out the ones that were out of focus.

### Statistical analysis

All experiments were repeated as three independent technical triplicates. The data were expressed as mean ± standard error of mean (SEM). Statistical comparisons were determined by unpaired t-test or one-way ANOVA with Tukey’s post hoc tests. p value less than 0.05 was defined as statistically significant. Statistical analyses were performed using GraphPad Prism Ver.6.0 (GraphPad Software Inc).

## Results

### Andrographis exhibits synergism with OPCs in enhancing anti-proliferative effects and inhibiting colony formation in colorectal cancer cells

**Figure 1:**
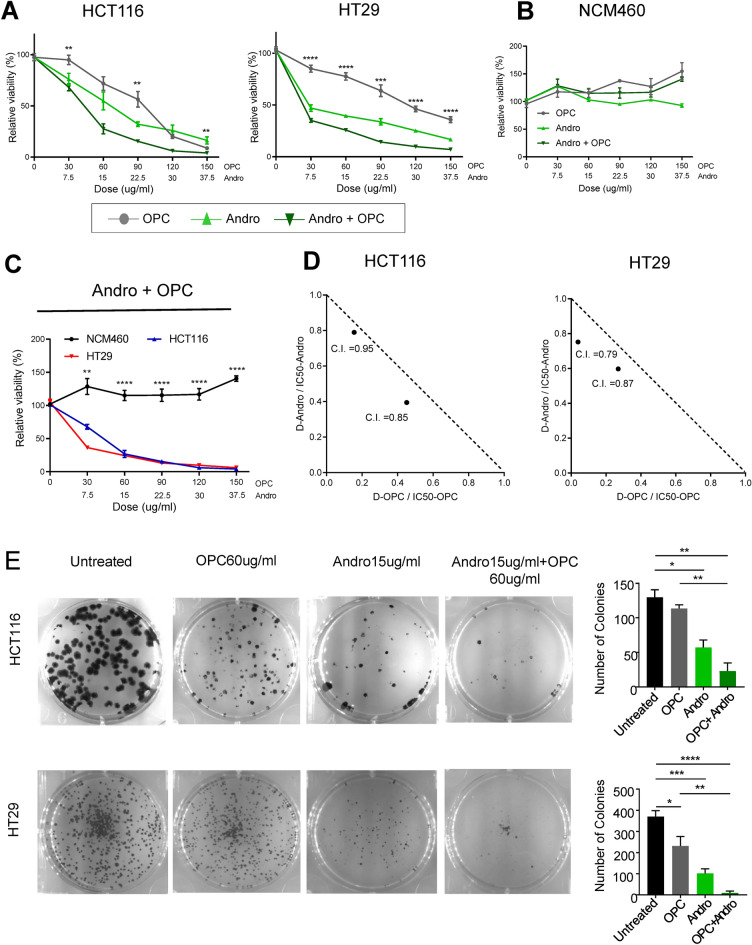
Andrographis and oligomeric proanthocyanidins (OPCs) enhance tumor suppressive effects in CRC cells. (**A**) Results of WST assay to compare cell viability following treatment with andrographis, OPCs and their combination for 72 h in HCT116 and HT29 cell lines. (**B**) Results of WST assay to compare cell viability following treatment with Andrographis, OPCs and their combination for 72 h in NCM460 normal colon epithelial cell. (**C**) Comparison of cell viability by WST assay following combination treatment between HCT116, HT29, and NCM460 cells. (**D**) Isobologram including Chou-Talalay combination index (CI) based on WST assay results to determine synergistic effects of andrographis and OPCs in HCT116 and HT29 cell lines. (**E**) Results of colony formation assay to assess clonogenicity of CRC cells following treatment with andrographis, OPCs, and the combination of both compounds for 7 days. Statistical significance is as follows; *P < 0.05, **P < 0.01, ***P < 0.001, and ****P < 0.0001 by one-way ANOVA test.

In order to evaluate whether andrographis has any synergistic activity in enhancing the efficacy of OPCs in CRC cells as reported previously^15, 17, 20^, we investigated the effects of both compounds individually (andrographis: 7.5, 15, 22.5, 30, 37.5 μg/ml; OPCs: 30, 60, 90, 120, 150 μg/ml;), as well as their combination, in HCT116 and HT29 cell lines. The WST assay demonstrated that andrographis exerted significantly greater growth inhibitory activity in all cell lines compared to OPCs. Furthermore, in support of our original hypothesis, the combination treatment with both compounds demonstrated significantly superior anti-proliferative effects in both cell-lines (Fig. 1A).

Subsequently, we also performed the WST assay using normal colon epithelial cells in order to evaluate the specificity of both, andrographis and OPCs treatments, alone or in combination. Interestingly, an equivalent dose-dependent treatment of andrographis and OPC used for HCT116 and HT29 when used in NCM460 cells, andrographis, OPC and the combination treatment did not induce cell toxicity to NCM460 cells (Fig. 1B,C) indicating that the anti-proliferative effect of these botanical compounds is specific only to cancer cells.

Interestingly, we observed that both andrographis and OPCs suppressed the growth proliferation of HCT116 and HT29 cell lines in a dose-dependent manner with the corresponding IC50 values of 19.0 and 10.0 μg/ml, respectively for andrographis and 85.9 and 55.7 μg/ml, respectively for OPCs. Furthermore, a combined treatment with both compounds further reduced the IC_50_ values of each botanical extract, as evidenced by the Chou–Talalay combination index (CI) that exhibited a synergistic efficacy between andrographis and OPCs in all cell lines (Fig. 1D), suggesting that andrographis with OPCs may enhance the anti-cancer effects of these compounds in CRC.

Next, we investigated the combinatorial effects of andrographis and OPCs on cell survival using the colony formation assay. Treatment of HCT116 and HT29 cells with andrographis or OPCs alone, drastically reduced the size of colonies compared with the corresponding controls (Fig. 1E). More importantly, the combination of these two compounds was more effective in inhibiting the colony formation compared with single treatment (Fig. 1E). Taken together, these data highlight the potential enhancement of anti-tumorigenic effect of both compounds when used in combination.

### Andrographis treatment potentiates the effects of OPCs through increased apoptosis in CRC cells

**Figure 2:**
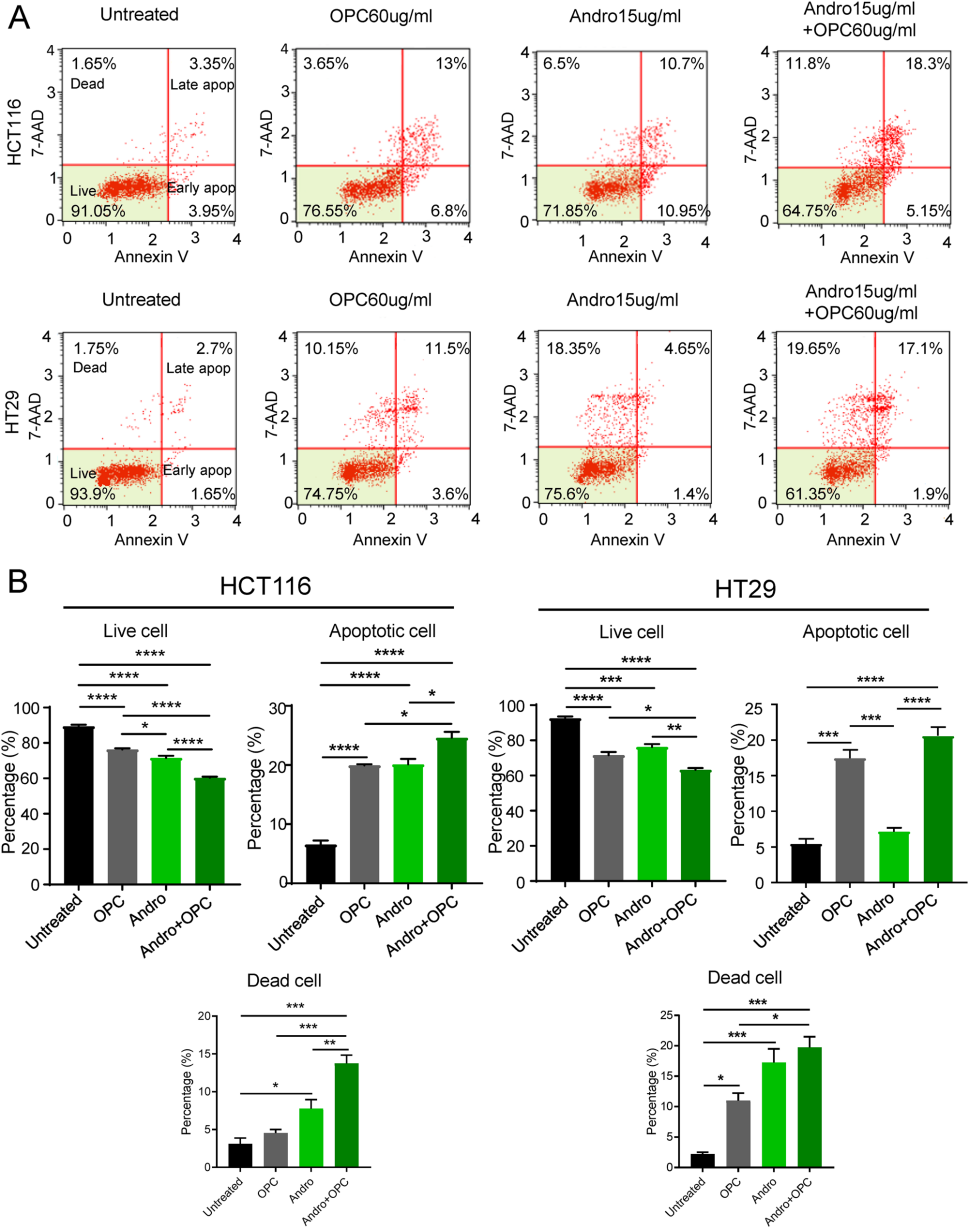
The combination of Andrographis and OPCs enhanced apoptotic activity in CRC cells. (**A**) Representative images illustrating percentage of cells undergoing apoptosis that stained positive for annexin-V assay in HCT116 and HT29 cells. The units are log10 transformed. (**B**) Bar graph shows the percentage of live cells and apoptotic cells of each treatment group in apoptosis assay. The assays were performed 48 h after treatment of each agent. Statistical Significance is as follows; *P < 0.05, **P < 0.01, ***P < 0.001, and ****P < 0.0001 by one-way ANOVA test.

Next, we were interested in knowing whether the reduced cell viability following the treatment with andrographis and OPCs was in part mediated by increased rates of apoptosis in CRC cells. Accordingly, we performed a series of experiments to evaluate the combinatorial impact of andrographis and OPCs on apoptotic rates via an Annexin V binding assay. It was quite reassuring to note that our results were consistent with our previous data for OPC-mediated increased rates of apoptosis; however, in this study we observed a similar effect with andrographis as well in CRC cell lines with a significant increase in HCT116 cells (p < 0.0001; Fig. 2A). More specifically, in comparison to untreated group, the combination treatment with andrographis and OPCs increased the percentage of apoptotic cells to 24.6% vs. 6.5% (p < 0.0001) and 20.6% vs. 5.4% (p < 0.0001) in HCT116 and HT29 cells respectively (Fig. 2B). More importantly, as was the case for reduced cell viability for the combination treatment with these two compounds, we observed that in fact the concurrent treatment with andrographis and OPCs further enhanced the apoptotic and dead cell population compared to single compound treatment, in both CRC cell lines (Fig. 2A,B). Taken together, these results suggest that andrographis potentiates the effects of OPCs through increased apoptosis and other cell-death related mechanisms in colon cancer cells.

### Andrographis mediated its anti-cancer activity through activation of genes involved in metabolic pathways and induction of ferroptosis

**Figure 3:**
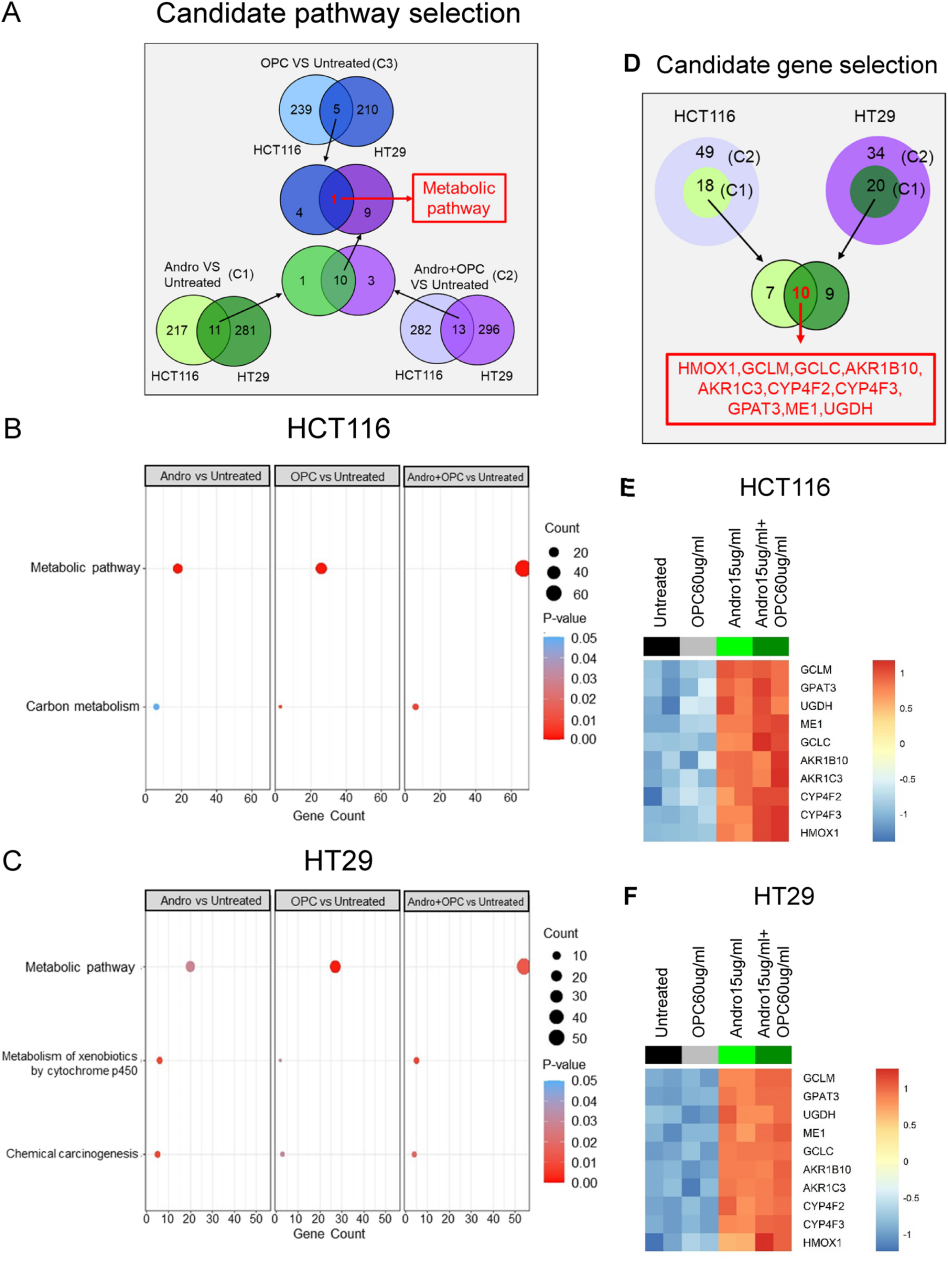
Genomewide transcriptomic profiling analysis. (**A**) Venn-diagram showing the number of dysregulated pathways in both cell-lines among different treatment group comparisons. (**B**,**C**) Dot plot showing the gene count of commonly dysregulated pathway in all comparison in HCT116 cells (B) and HT29 cells (C). (**D**) Venn-diagram showing the selection process of candidate genes. (**E**,**F**) Heatmap showing the expression profile of candidate genes in different treatment groups of HCT116 (**E**) and HT29 cells (**F**). Red to blue colors on the heatmap represent relatively high to low log normalized gene expression values.

Next, to decipher the molecular mechanisms underlying the anti-tumorigenic properties of andrographis and combination treatment, we performed genome-wide transcriptomic profiling of HCT116 and HT29 cells in untreated cells as well as cells treated with andrographis, OPCs and their combination. Following expression profiling of these cell lines, we performed Gene Set Enrichment Analysis on significantly dysregulated genes, which led us to identify multiple pathways which were significantly dysregulated in individual as well as combination treatment groups in both CRC cell lines (Fig. 3A, Supplementary Fig. S1A). Upon further investigation, we observed that not only significant enrichment of metabolic pathways was common between all three treatment groups (Fig. 3A, Supplementary Fig. S1A) but was also higher in the combination group with regard to dysregulated gene count (Fig. 3B,C). These findings indicated that the enhanced anti-cancer activity of the combination treatment is perhaps mediated through the enhanced activation of metabolic pathways. Subsequent selection process of candidate metabolic pathway related genes led to the identification of HMOX1, GCLC, GCLM, AKR1B10, AKR1C3, CYP4F2, CYP4F3, GPAT3, and ME1 as candidate metabolic pathway-related genes which were significantly and commonly dysregulated in HCT116 and HT29 cells (Fig. 3D–F, Supplementary Table S2). The candidate genes, HMOX1, GCLC, and GCLM were not only part of the metabolic pathway but are also involved in ferroptosis pathway.

**Figure 4:**
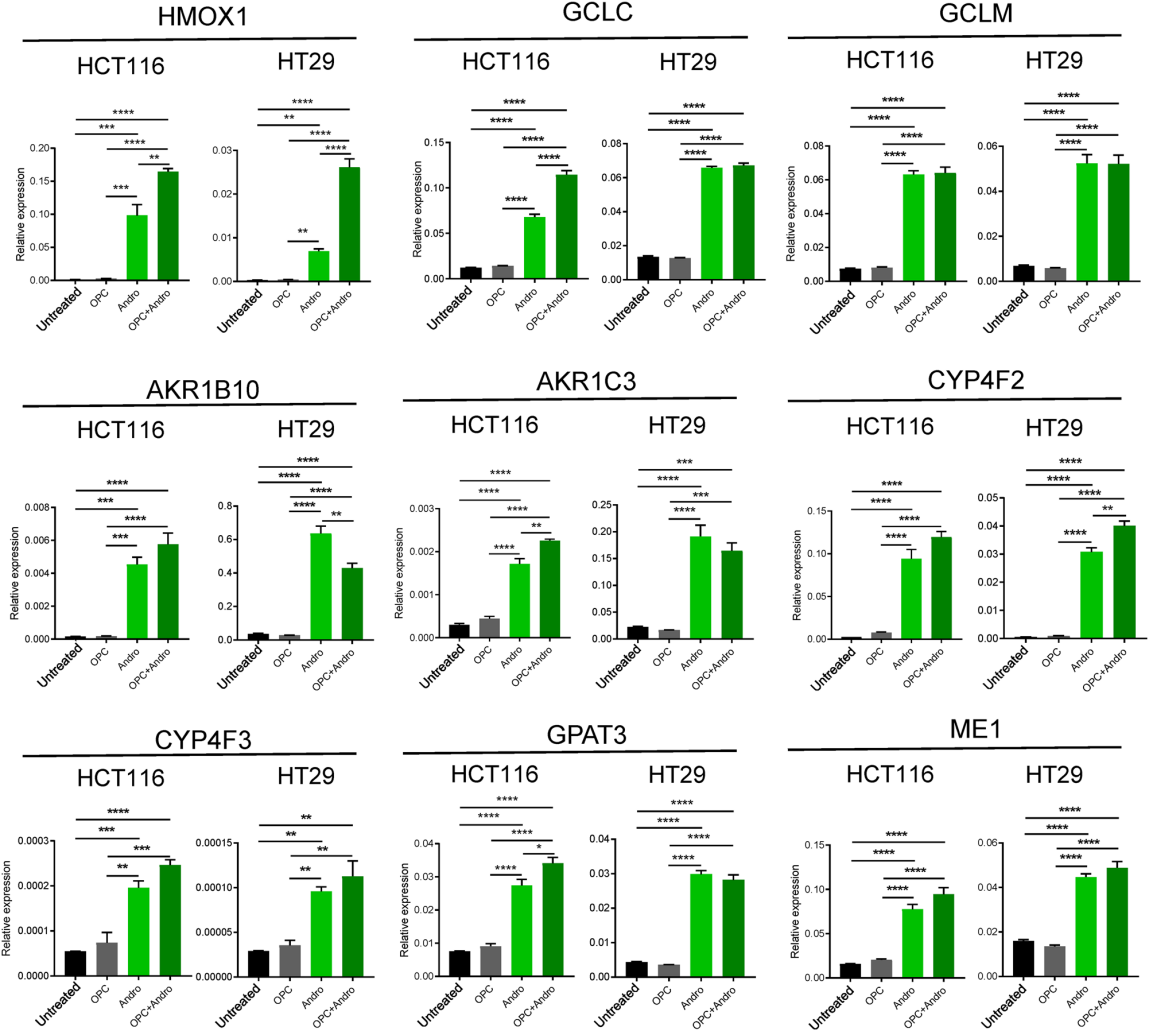
Andrographis up-regulates metabolic pathway and ferroptosis-related genes in CRC cells. Alterations in mRNA expression of metabolic pathway and ferroptosis-related genes viz. HMOX1, GCLC, GCLM, AKR1B10, AKR1C3, CYP4F2, CYP4F3, GPAT3 and ME1 following andrographis, OPCs, and the combination treatment for 16 h in HCT116 and HT29 cells. Statistical Significance is as follows: *P < 0.05, **P < 0.01, ***P < 0.001, and ****P < 0.0001 by one-way ANOVA test.

To further confirm the findings from our gene expression profiling studies, we performed validation studies using RT-qPCR. All target genes including AKRB1B10 (p < 0.0001); AKR1C3 (p < 0.0001); CYP4F2 (p < 0.0001); CYP4F3 (HCT116 p < 0.001; HT29 p < 0.01), GPAT3 (p < 0.0001), ME1 (p < 0.0001), HMOX1 (p < 0.0001), GCLC (p < 0.0001) and GCLM (p < 0.0001) were significantly upregulated after andrographis or combination treatment compared to untreated group at mRNA levels in both cell lines (Fig. 4). In particular, HMOX1, which is a critical mediator of metabolic and ferroptosis induction, was most significantly upregulated gene in response to treatment with the combination treatment (p < 0.0001), when compared to either OPC treated cells or untreated cells, in both the cell lines. HMOX1 was identified as most upregulated target by both expression profiling and RT-qPCR analysis. Taken together, these results indicate activation of ferroptosis and metabolic pathways as possible mechanisms that might be contributing to the enhanced anti-tumorigenic properties of andrographis and OPCs in colon cancer cells.

### A combined treatment with Andrographis and OPCs exhibits a substantial tumor suppressive effect in a xenograft animal model

**Figure 5:**
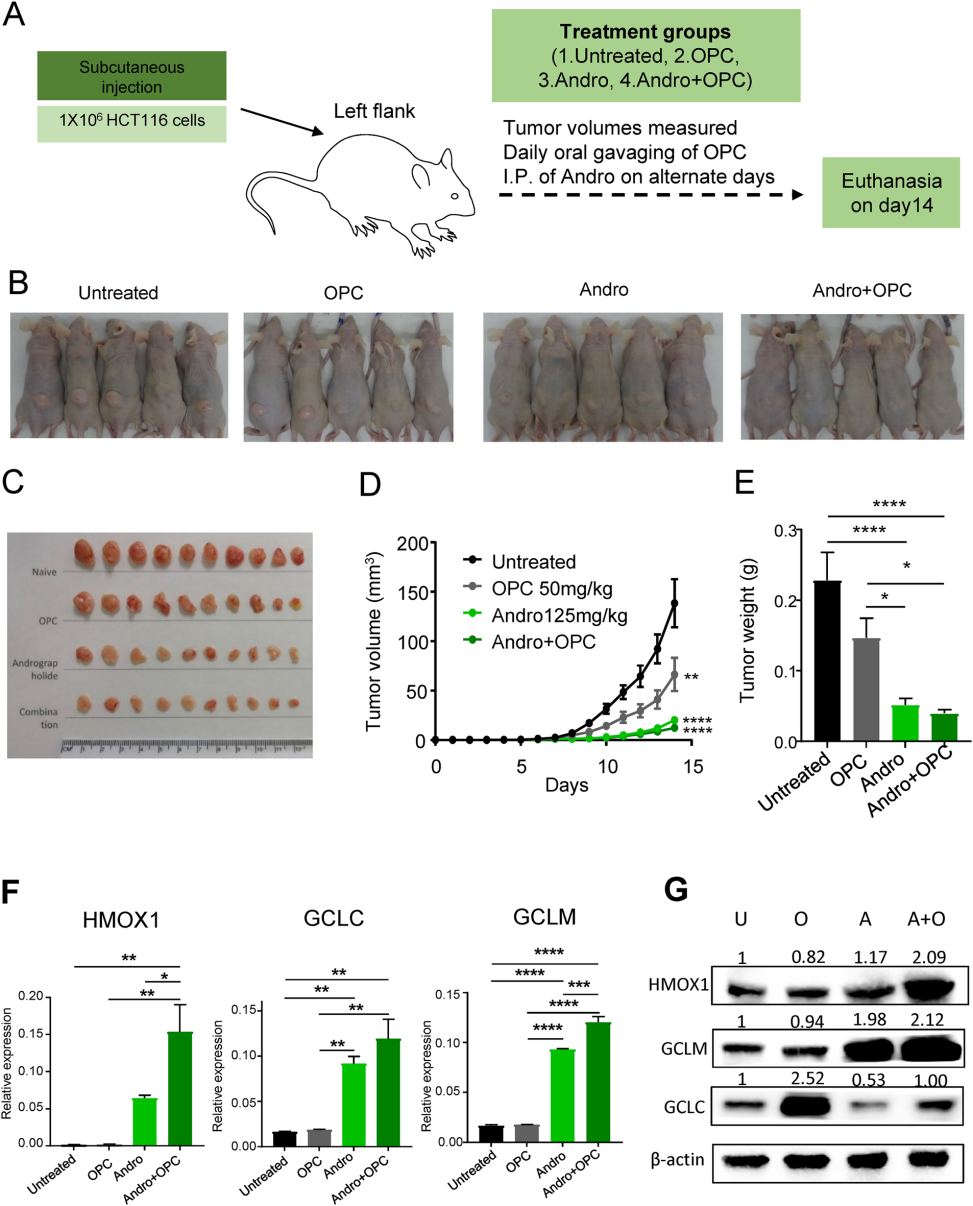
Andrographis suppresses tumor growth in a xenograft animal model. (**A**) Schematic for the establishment of HCT116 cell line-derived xenograft in athymic mice, and the treatment schedule of andrographis and OPCs. (**B**) Representative image of xenograft tumor in each treatment group. (**C**) Tumor volume alterations in each treatment group. (**D**) Representative images of harvested tumors in each treatment group. (**E**) Bar graphs of harvested tumor weight in each treatment group. (**F**) Bar graphs showing mRNA expression of ferroptosis-related genes (HMOX1, GCLC and GCLM) of each treatment group in xenograft tumors. Statistical Significance is as follows: *P < 0.05, **P < 0.01, ***P < 0.001, and ****P < 0.0001 by one-way ANOVA test. (**G**) Alterations in protein expression levels of HMOX1, GCLC and GCLM following andrographis, OPCs, and combination treatment in tumor xenografts. Beta-actin was used as a loading control. Full-length blots are presented in Supplementary Fig. S3.

In order to further validate our in-vitro results, we next investigated the anti-tumorigenic effects of andrographis and OPCs in an animal model, by following the tumor growth kinetics in a subcutaneous xenograft mice model. We generated CRC xenografts by subcutaneous injection of 1 × 10^6^ HCT116 CRC cells. Approximately five days following the subcutaneous injection on the abdominal flanks of mice, the tumors became palpable. Thereafter, we segregated these animals into four groups; untreated group, OPCs treatment (daily oral gavage of 50 mg/kg of OPCs), andrographis treatment (intraperitoneal injection of 125 mg/kg andrographis on alternate days) and the combination treatment (treatment with both andrographis and OPCs). Finally, the mice were sacrificed on day 14 after subcutaneous injection (Fig. 5A).

Although the daily tumor volume in untreated group increased exponentially, tumor volumes were significantly suppressed in animals treated with OPCs (p < 0.01). However, the treatment with andrographis exhibited a significantly stronger tumor suppressive effect individually, as well as in the combination treatment group, vis-a-vis, the other two groups (p < 0.0001, respectively; Fig. 5B,C). With regards to the collected tumor weight on day 14, the results were consistent with the tumor volume findings, wherein the tumor weight of andrographis treatment group and combination treatment group was significantly lower compared to the untreated group (p < 0.0001, respectively; Fig. 5D,E).

Next, we extracted RNA from harvested xenograft tumors and investigated the expression of metabolic pathway and ferroptosis associated genes. All three candidate genes, including HMOX1, GCLC, and GCLM, were significantly up-regulated in the andrographis and the combination treatment group vs. the untreated group (p < 0.0001 to < 0.01), which were consistent with our in-vitro experiments (Fig. 5F). Further validation by western blot analysis showed two folds increase in expression of HMOX1 and GCLM in combination group as compared to the untreated or OPC treatment group (Fig. 5G). On the other hand, among metabolic pathway associated genes, the expression of AKR1B10 and CYP4F3 was significantly upregulated in the combination treatment group vs. the untreated group (p < 0.001 to < 0.01; Supplementary Fig. S2). Collectively, these data demonstrate that while the tumor regressive effect of combination treatment is mainly contributed by andrographis treatment in the xenograft model, the genotypic alteration of the tumor tissue is slightly more affected in combination treatment group than in untreated and single agent treatment group, and ferroptosis and metabolic pathway related genes could possibly be associated with such an effect.

### Andrographis treatment potentiated the antitumor effect of OPCs and inhibited tumor growth in patient derived primary epithelial organoids

**Figure 6:**
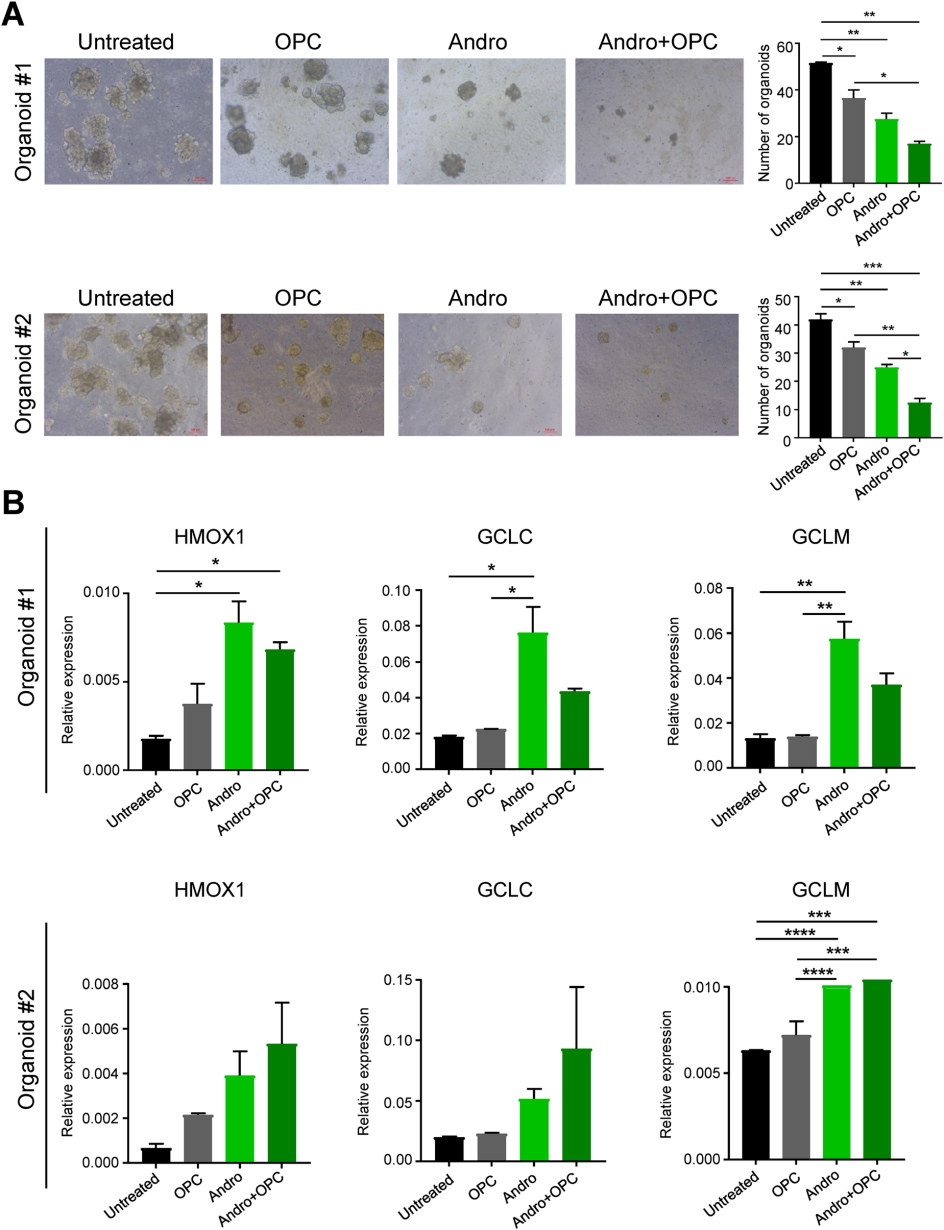
Andrographis and combination treatment with OPCs enhanced tumor growth suppression of organoids derived from human colorectal tumors. (**A**) Representative images of tumor organoid cultures in each treatment group and the bar graphs showing the number of organoids in each treatment group. (**B**) Bar graphs illustrate mRNA expression of ferroptosis-related genes (HMOX1, GCLC, and GCLM) of each treatment group in CRC tumor organoid derived from two different patients. Statistical Significance is as follows: *P < 0.05, **P < 0.01, ***P < 0.001, and ****P < 0.0001 by one-way ANOVA test.

Finally, to better appreciate the significance of our data, we next established tumor-derived primary epithelial 3D organoids derived from CRC patients and examined the tumor-suppressive effects of andrographis and OPCs. Organoid cultures derived from human CRC specimens were treated with andrographis, OPCs and their combination for a week. In line with our other experimental results, the combination treatment with andrographis and OPCs significantly decreased the growth and number of patient-derived tumor organoid formation in comparison to vehicle treated group (p < 0.01 for organoid 1, and p < 0.001 for organoid 2) and the OPCs treatment group (p < 0.05 for organoid 1, and p < 0.01 for organoid 2; Fig. 6A). Although there was significant reduction (50%) in number of organoids in combination treatment group as compared to andrographis alone for organoid #2, only a moderate (38.2%) reduction was seen for organoid#1. Furthermore, upregulation of ferroptosis pathway related gene was also modest in combination compared to Andrographis alone treatment group for organoid #1 in contrast to organoid #2 (Fig. 6B). This difference could be due to inter tumor heterogeneity. Of note gene expression profiles of organoids faithfully represent the gene expression profiles of tumors they are derived from^30^, and CRC has been reported to exhibit molecular heterogeneity^31^. These results suggest that molecular heterogeneity of tumors from different patients could affect the efficacy of this combination.

## Discussion

CRC is a common malignancy in the western countries, and hundreds and thousands of lives are lost to this disease globally each year. Intrinsic and acquired resistance to conventional chemotherapeutic drugs is one of the biggest challenges in improving therapeutic outcomes in CRC. There is a growing consensus that a small pool of cancer cells that are resistant to any targeted agent are often present in solid tumors at the initiation of therapy, and these resistant clones of cells continue to clonally expand once the therapy is administered leading to disease relapse^5^. This leads to a significant clinical challenge as therapies targeting single targets often have limited therapeutic success, highlighting the importance of the use of drug combinations that can improve therapeutic response by targeting multiple pathways and inhibiting evolution of resistant cancer cells^6^. However, the anti-cancer benefits of using drug combination are often offset by the simultaneous increase in drug toxicity and the expense associated with such treatment modalities. In this context, with an untapped structural diversity and time-tested safety and efficacy, dietary compounds offer a safe and cost-effective approach. Notably, in the area of cancer therapeutics, over the time frame of 1940s to 2014, of the 175 small molecules used as anti-cancer drugs, 85 of them were either natural products or direct derivatives from such natural remedies^32^; highlighting the medicinal importance and safety profiles of these dietary botanicals. More specifically, most of these botanicals also work by targeting multiple pathways and interact together as for anti-cancer effects on tumor growth, making them attractive propositions for reducing drug resistance and improving the overall therapeutic efficacy. Based upon our previous studies on OPCs from the grape seed extracts^10, 15, 17, 20^, herein we interrogated whether a combination of andrographis and OPCs might demonstrate superior anti-tumorigenic properties in CRC- by examining its effects in cell lines, xenograft animal models and patient-derived organoids.

Accumulating evidence has demonstrated additional effects of andrographis with some of the conventional chemotherapeutic drugs. For instance, andrographis potentiated the antitumor effects of topotecan, paclitaxel, cisplatin and gemcitabine in acute myeloid leukemia, non-small cell lung cancer, oral squamous cell carcinoma and pancreatic cancer, respectively^33–36^. Previous studies have also demonstrated the anti-proliferative and chemo-sensitizing properties of andrographis in CRC cells^37, 38^. In this study, by performing a series of experiments in various in-vitro and ex-vivo model systems, we for the first time established the substantially enhanced anti-tumor properties of andrographis when combined together with OPCs, and also identified the possible molecular pathways through which andrographis and OPCs potentially exert their anticancer properties. Our results indicated an enhanced growth inhibitory activity of andrographis and OPCs with a significantly decreased IC_50_ value in CRC cells when used in combination as compared to single-agent treatments. In addition, the combination of andrographis and OPCs decreased colony formation potential of CRC cells, increased apoptosis, and reduced growth of tumors in CRC xenografts. Although organoid culture recapitulates the physiological properties of the primary tissue including tumor heterogeneity^30, 39^ and the effectiveness of the compounds varied between different organoid, combination treatment also tended to show its tumor-regressive effect in the organoid study.

Previous studies have revealed that the anti-tumorigenic effects of andrographis in CRC cells were associated with the suppression of the TLR4/NF-κB/MMP-9/NADPH Oxidase and Src/MAPKs/AP-1 axis and through enhancement of the expression of the BAX protein^37, 40, 41^. However, until now, no efforts have been elucidated the effect of andrographis on genomewide expression profiles in CRC. Accordingly, by using genome-wide expression profiling in this study, we revealed that andrographis and OPCs commonly affected several growth regulatory pathways, leading to a more effective tumor growth suppression in cancer cells. By using qRT-PCR and western blot analysis, we demonstrated that treatment of andrographis combined with OPCs enhanced their anti-cancer activity possibly through increased metabolic pathway and induction of ferroptosis, however further mechanistic validation is warranted. Ferroptosis is a process that is referred to as programmed necrosis which is primarily induced by extra-mitochondrial lipid peroxidation derived from an iron-dependent ROS accumulation^42^. At transcript level, we observed upregulation of HMOX1, GCLC and GCLM by andrographis. These results are in line with previous studies where treatment of andrographis enhanced the expression of HMOX1 and GCLM to support its anti-tumor activity^36, 43^. Of note, it is well established that transcription from promoters of all three genes are controlled by antioxidant response element (ARE) and the up-regulation of HMOX1 and GCLM induced by andrographis might be through altered PI3K/Akt signaling pathway^44^. In our study, andrographis treatment enhanced the expression of HMOX1 in combination with OPC. Considering the fact that HMOX1 is a positive regulator of ferroptosis^45^, our data suggests the possible activation of ferroptosis in the combination treatment. With regards to other metabolic pathway associated genes, AKR1B10 which is a metabolic enzyme that catalyzes aldo–keto reduction, has also received a great deal of attention in terms of its relationship with the p53 gene regulation in CRC^46^. It was demonstrated that AKR1B10 was identified as a direct target of p53 family and the overexpression of AKR1B10 enhanced p53-induced apoptosis and inhibited tumor proliferation in CRC cell-line and xenograft model^46^. Furthermore, another study revealed that CRC patients with high expression of AKR1B10 showed better prognosis^47^. Taken together, from our genome-wide comprehensive expression profiling data in CRC cells with andrographis and combination treatment, sheds light on the mechanistic insight of these novel therapeutic targets and the positive strong effect to them by andrographis or combination treatment in CRC.

We would like to acknowledge some of the limitations of our current study. First, our in-vitro study consists of just two cell-lines, and none of them are RAS/BRAF wild type, which encompass the majority of CRC. Second, with regard to the xenograft study, tumor regressive effect in the combination treatment group were apparently more influenced by Andrographis than by OPC. Third, as for the organoid study, expression of ferroptosis and metabolic pathway related genes was significantly altered in one of the two organoids. These differences in the gene expression between the two organoids might be due to the inherent tumor heterogeneity between the organoids. Lastly, since the primary purpose of this study was to identify key pathways involved in the anti-cancer activity of Andrographis and OPCs, we did not perform detailed mechanistic studies on ferroptosis pathways. Therefore, further studies are warranted to further characterize and dissect the molecular underpinnings of these dietary botanicals on ferroptosis and metabolic pathways.

Collectively, in addition to providing another layer of support for their enhanced anti-tumorigenic activity, this is the first report highlighting the activation of metabolic pathway and ferroptosis as one of the possible mechanisms that contributes to the anti-tumorigenic and interactive properties of andrographis and OPCs from the grape seed extract, therefore making a case for their use as an adjunctive treatment in colorectal cancer.

## Conclusions

In conclusion, we have demonstrated that combined treatment with andrographis and OPCs enhanced the tumor suppressive effects in CRC possibly through the activation of metabolic pathway and enhanced ferroptosis however further mechanistic validation is warranted. As targeting of metabolic pathways and ferroptosis are one of the promising therapeutic strategies in human cancer, our study may provide essential evidence in support of the combinational use of andrographis and OPCs as a potential therapeutic option for exploration in further studies, perhaps as an adjunctive treatment to classic drugs, in patients with colorectal neoplasia.

## Supplementary Information

Supplementary Information 1.

Supplementary Information 2.
