# Supplementary material for: Enhanced anti-cancer activity of andrographis with oligomeric proanthocyanidins through activation of metabolic and ferroptosis pathways in colorectal cancer
Source: Sci Rep. 2021 Apr 6;11:7548. doi: 10.1038/s41598-021-87283-y (PMC8024269; doi:10.1038/s41598-021-87283-y)
Supplement: Supplementary file 1 — Supplementary Information 1. [file 41598_2021_87283_MOESM1_ESM.docx]

**Supplementary Figure 1:** Significantly dysregulated pathway between treated vs untreated cells. Dot plot showing the gene count of the significantly dysregulated pathway between, 1.OPC vs untreated, 2.Andrographis vs untreated, and 3.Combination vs untreated groups both in HCT116 cells and HT29 cells.

**Supplementary Figure 2:** Andrographis or combination treatment affect the expression of metabolic pathway associated genes in a xenograft animal model. Bar graphs showing mRNA expression of metabolic pathway related genes (AKR1B10, CYP4F3, AKR1C3, CYP4F2, GPAT3, and ME1) of each treatment group in xenograft tumor. Statistical Significance is as follows: *P < 0.05, and **P < 0.01 by one-way ANOVA test.

**Supplementary Figure 3:** Effect of Andrographis treatment or combination treatment on metabolic and ferroptosis pathways. Uncropped western blot images of the selected metabolic and ferroptosis pathway genes are shown.
