# Supplementary material for: Enhanced anti-cancer activity of andrographis with oligomeric proanthocyanidins through activation of metabolic and ferroptosis pathways in colorectal cancer
Source: Sci Rep. 2021 Apr 6;11:7548. doi: 10.1038/s41598-021-87283-y (PMC8024269; doi:10.1038/s41598-021-87283-y)

Enhanced anti-cancer activity of andrographis with oligomeric proanthocyanidins through activation of metabolic and ferroptosis pathways in colorectal cancer

Tadanobu Shimura^1*^, Priyanka Sharma^1,2*^, Geeta G. Sharma^2*^, Jasjit K Banwait^1^, and Ajay Goel^1,2^

^1^ Center for Gastrointestinal Research, Baylor Scott & White Research Institute and Charles A. Sammons Cancer Center, Baylor University Medical Center, Dallas, TX, USA.

^2^ Department of Molecular Diagnostics and Experimental Therapeutics, Beckman Research Institute, City of Hope Comprehensive Cancer Center, Duarte, CA, USA.

* Authors contributed equally.

**SUPPLEMENTARY INFORMATION**

We show the information of supplementary Table1, supplementary Table2, supplementary Figure 1, supplementary Figure 2, and supplementary Figure 3.

**Supplementary Table 1: List of primers used in this study**

| HMOX1 | Fwd | AAGACTGCGTTCCTGCTCAAC |
| --- | --- | --- |
|  | Rev | AAAGCCCTACAGCAACTGTCG |
| GCLC | Fwd | AGGCCAACATGCGAAAAC |
|  | Rev | CGGATATTTCTTGTTAAGGTACTGG |
| GCLM | Fwd | GGGGAACCTGCTGAACTG |
|  | Rev | AGATACAGTGCATTCCAAGACATC |
| AKR1B10 | Fwd | AAAGCAACGTTCTTGGATGC |
|  | Rev | TGGAAGTGGCTGAAATTGG |
| AKR1C3 | Fwd | CATTGGGGTGTCAAACTTCA |
|  | Rev | CCGGTTGAAATACGGATGAC |
| CYP4F2 | Fwd | CAGCCTCATGACCTTGGACA |
|  | Rev | GCTCCAAGATGGCGGCAAT |
| CYP4F3 | Fwd | ATTGGTTCTTGGGTCACCTG |
|  | Rev | GCCAGGCTTTGTGTGTATAGG |
| GLA | Fwd | CTCCTGTGAGTGGCCTCTT |
|  | Rev | ATTTCGCCAGTGATTGCAGT |
| GPAT3 | Fwd | GGTGCTGGGCGTCATAGT |
|  | Rev | CCCAATGAAAGCCAAGGTAA |
| ME1 | Fwd | GCAGTGCTACAAAATAACCAAGG |
|  | Rev | TGGAAGAGTGACTGGATCAAAA |
| B-actin | Fwd | CATGTACGTTGCTATCCAGGC |
|  | Rev | CTCCTTAATGTCACGCACGAT |

**Supplementary Table2:** Process of target gene selection

| HCT116 | | | | HT29 | | | | HCT116 | HT29 |
| --- | --- | --- | --- | --- | --- | --- | --- | --- | --- |
| Andro vs Untreated | Comb vs Untreated | | | Andro vs Untreated | Comb vs Untreated | | | Common | Common |
| HMOX1 | HMOX1 | SC5D | P4HA2 | AKR1C3 | DHRS9 | NAMPT | CYP24A1 | HMOX1 | AKR1C3 |
| CYP4F3 | CYP4F3 | TUSC3 | FASN | AKR1B10 | AKR1C3 | GCNT2 | ACSL1 | CYP4F3 | AKR1B10 |
| GCLM | GCLC | FDPS | ELOVL6 | GPAT3 | CYP4F3 | UGDH | PCYT1A | GCLM | GPAT3 |
| GCLC | CYP4F2 | CYP51A1 | NSDHL | CYP4F3 | HMOX1 | GBE1 | GALNT5 | GCLC | CYP4F3 |
| CYP4F2 | GCLM | G6PD | IDS | ALDH3A1 | GPAT3 | GLA | GALNT8 | CYP4F2 | ALDH3A1 |
| AKR1B10 | AKR1B10 | ALAS1 | TM7SF2 | HMOX1 | AKR1B10 | TBXAS1 | ITPKC | AKR1B10 | HMOX1 |
| ME1 | ALDOC | DHCR24 | CHPF2 | GCLM | GCLM | ST3GAL1 | INPP1 | ME1 | GCLM |
| AKR1C3 | MSMO1 | UGDH | MGAT5 | GCLC | LAMA3 | ALDOC | UPP1 | AKR1C3 | GCLC |
| GPAT3 | IDI1 | CHAC1 | PCYT2 | LAMA3 | ALDH3A1 | SAT1 | CHST8 | GPAT3 | LAMA3 |
| GLA | ME1 | HPD | AGPAT4 | CYP4F2 | GCLC | CDA | HPD | GLA | CYP4F2 |
| ALAS1 | LPIN1 | ITPKC | GBE1 | PGD | CYP4F2 | P4HA1 | RDH10 | ALAS1 | PGD |
| G6PD | ACSS2 | PGD | PHYKPL | HGD | LPIN1 | FECH | SYNJ2 | G6PD | HGD |
| P4HA1 | GPAT3 | BLVRB | TKT | CBR3 | PGD | MSMO1 | ST6GALNAC3 | P4HA1 | CBR3 |
| UGDH | HSD17B7 | LIPG | ACSL4 | IDS | IDS | AGPAT4 | B4GALT6 | UGDH | IDS |
| B4GALNT1 | HMGCR | B4GALNT1 | SGMS2 | HKDC1 | HGD | G6PD |  | B4GALNT1 | HKDC1 |
| TUSC3 | GLA | MVK | GDA | ME1 | CBR3 | CBR1 |  | TUSC3 | ME1 |
| ALDH3A2 | P4HA1 | HSD17B12 | GFPT1 | GLA | ME1 | RIMKLA |  | ALDH3A2 | GLA |
| HPD | DHCR7 | ASNS | RDH11 | GBE1 | HKDC1 | PLPP3 |  | HPD | GBE1 |
|  | AKR1C3 | ALDH3A2 | NDUFA10 | NAMPT | ACSS2 | PFKP |  |  | NAMPT |
|  | ACAT2 | MMAB | EBP | UGDH | NT5E | ELOVL1 |  |  | UGDH |
|  | FDFT1 | PNPLA3 | OGDH |  |  |  |  |  |  |
|  | MVD | FECH |  |  |  |  |  |  |  |
|  | SQLE | LPIN1 |  |  |  |  |  |  |  |

Process 1. Light-green highlighted genes are significantly dysregulated in both (1) Andro-treatment vs Untreated group, and (2) Combined treatment vs Untreated group in HCT116 cells.

Process 2. Dark-green highlighted genes are significantly dysregulated in both (1)Andro-treatment vs Untreated group, and (2) Combined treatment vs Untreated group in HT29 cells.

Process 3. Red highlighted genes are significantly dysregulated genes both in Process 1 and Process 2.

**Supplementary Figure1**


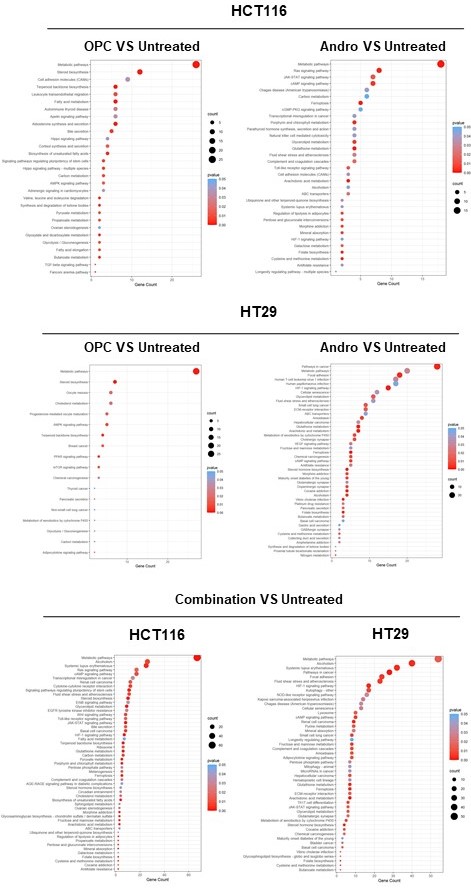


**Supplementary Figure2**


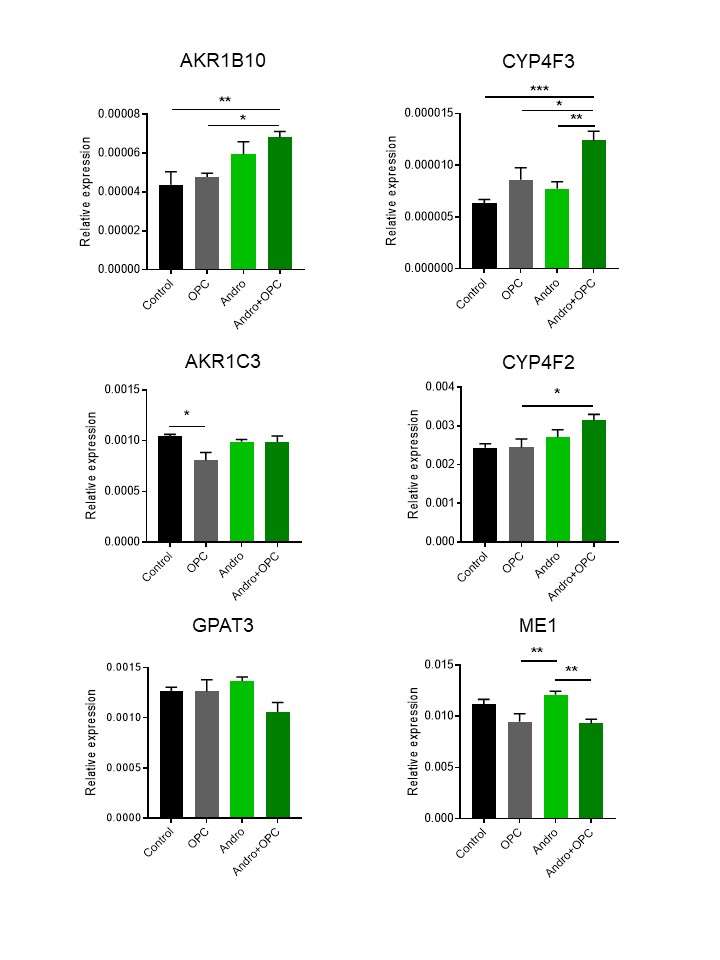


**Supplementary Figure3**


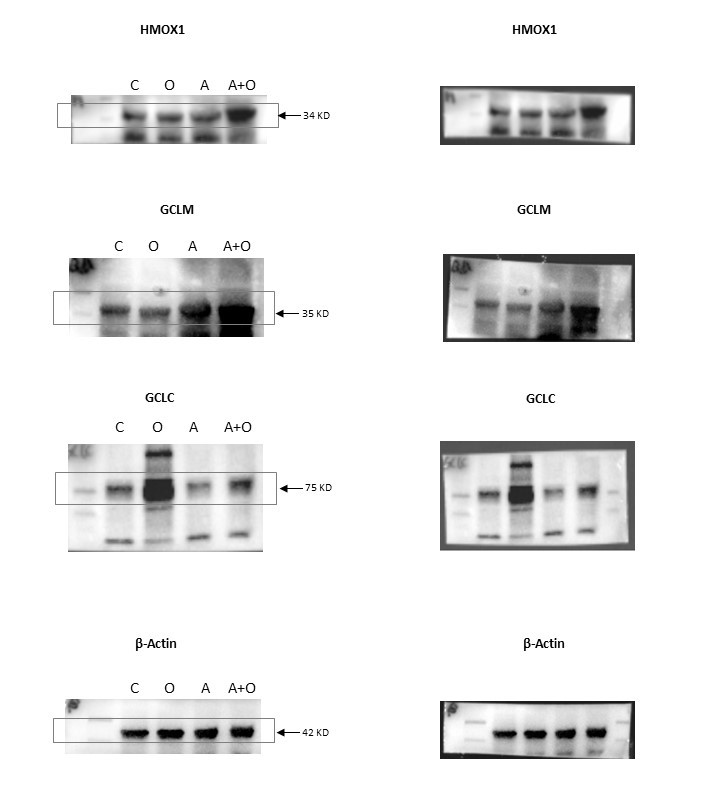

Supplement: Supplementary file 2 — Supplementary Information 2. [file 41598_2021_87283_MOESM2_ESM.docx]
